# Depth-Camera-Aided Inertial Navigation Utilizing Directional Constraints

**DOI:** 10.3390/s21175913

**Published:** 2021-09-02

**Authors:** Usman Qayyum, Jonghyuk Kim

**Affiliations:** 1 Center of Excellence in Science & Applied Technology (CESAT), Islamabad 45550, Pakistan; mrusmanqayyum@gmail.com; 2 Robotics Institute, University of Technology Sydney, Sydney, NSW 2006, Australia

**Keywords:** integrated inertial navigation, depth camera, directional constraints, epipolar constraints

## Abstract

This paper presents a practical yet effective solution for integrating an RGB-D camera and an inertial sensor to handle the depth dropouts that frequently happen in outdoor environments, due to the short detection range and sunlight interference. In depth drop conditions, only the partial 5-degrees-of-freedom pose information (attitude and position with an unknown scale) is available from the RGB-D sensor. To enable continuous fusion with the inertial solutions, the scale ambiguous position is cast into a *directional constraint* of the vehicle motion, which is, in essence, an epipolar constraint in multi-view geometry. Unlike other visual navigation approaches, this can effectively reduce the drift in the inertial solutions without delay or under small parallax motion. If a depth image is available, a window-based feature map is maintained to compute the RGB-D odometry, which is then fused with inertial outputs in an extended Kalman filter framework. Flight results from the indoor and outdoor environments, as well as public datasets, demonstrate the improved navigation performance of the proposed approach.

## 1. Introduction

Autonomous small-scale aerial vehicles such as drones have drawn significant attention from academia and industry due to their accessibility, low cost, and easy operation, with many potential applications. A continuous and robust navigation solution is crucial for these vehicles to perform automatic control and guidance. To operate in cluttered environments or in proximity to environments where the Global Navigation Satellite System (GNSS) signals can be partially or fully blocked, various perception sensors (e.g., laser scanners or cameras) are incorporated for odometry or simultaneous localization and mapping (SLAM) solutions.

Due to the lightweight and rich information, the camera-based system has been actively researched for small-scale aerial vehicles. In particular, affordable, consumer-grade RGB-D cameras (providing color and depth, such as Microsoft Kinect and RealSense) have enabled considerable advancement for 3D reconstruction and SLAM odometry navigation [1,2,3,4]. Although quite successful, most current applications have been limited to indoor scenarios due to the limited sensing range and depth dropout problems. The presence of strong infrared interference from the sunlight significantly reduces the maximum depth range (less than 4m in typical outdoor conditions). In addition, aerial vehicles typically require enough clearance from the environment to avoid any collision and operate safely. Consequently, the RGB-D sensor would act virtually as a monocular camera, causing a depth dropout problem, limiting the usability of RGB-D sensors in outdoor flying conditions.

**Figure 1:**
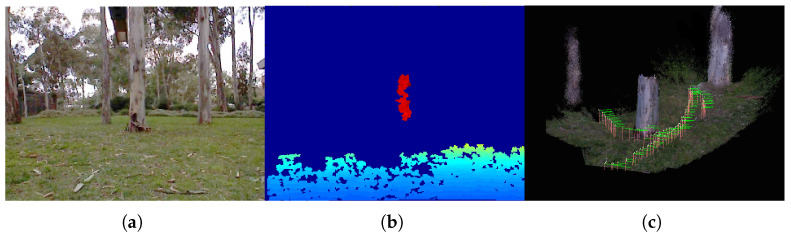
(**a**) A Kinect color image from an aerial vehicle, (**b**) showing partial depths from a tree trunk and the ground. (**c**) Reconstructed 3D map utilizing the direction constraints proposed in this work.

Figure 1 shows typical RGB-D images collected from an aerial vehicle, showing partial or no depth images. It also shows a reconstructed 3D map and trajectory obtained from this work. In addition, aerial vehicles typically experience a high rotational rate and/or acceleration during maneuvers. For example, a high angular motion of the vehicle but with small parallax can make the triangulation process slow and difficult. A high dynamic sensor, such as an inertial measurement unit (IMU), is required to track the motion and features. In the IMU-aided visual navigation system, the challenge occurs when the RGB-D sensor degenerates to the monocular mode. The scale-ambiguous (non-metric) visual translation needs to be fused with the (metric) inertial output. Although the scale can be estimated from the inertial navigation system, the unaided low-quality inertial sensor cannot converge until the features are robustly initialized.

This work addresses the depth dropout problem by proposing a novel Inertial-RGB-D (Kinect) fusion method that effectively integrates the inertial odometry outputs and RGB-D or monocular images. The contributions of this work are as follows:

- The use of the *directional constraint* of the non-metric visual translation to aid the inertial solutions. It is based on our preliminary work [5], providing more thorough results using a public dataset as well as outdoor experiments.
- Our Inertial-Kinect odometry system integrates the full 6 degrees of freedom (DOF) (rotation and translation) and partial 5DOF (rotation and scale-ambiguous translation) information from the Kinect to estimate the pose of an aerial vehicle. Most existing works have been directed at indoor applications in which the full 6DOF Kinect poses are available.
- We demonstrate real-time, front-end odometry while the back-end pose-graph SLAM supports low-priority multi-threaded processing for the keyframe optimization. The real-time odometry outputs are subsequently used for hovering flight control in a cluttered outdoor environment.

This directional constraint essentially comprises the epipolar constraints of features between a pair of images that can aid an inertial system [6,7], and recently more computationally efficient multistate-constraint filters [8,9]. Although we rely on the same epipolar principle (actually any visual ego-motion method relies on this constraint), our method is different in that we cast the epipolar constraint as the directional constraint of the vehicle motion, which is not limited to a planar scene or estimating the epipolar points. The key benefit is the undelayed aiding of the IMU solution even under low parallax motion. In addition, our method does not require the popular inverse depth parameterization, which requires augmented state dimensions, thus more computational complexity.

If monocular configuration is used all the time, for example, due to the extended period of depth-dropout, the performance will be similar to the standard visual odometry method, causing scale drift over time along the direction of the motion. The tangential direction error can be limited from the directional fusion.

**Figure 2:**
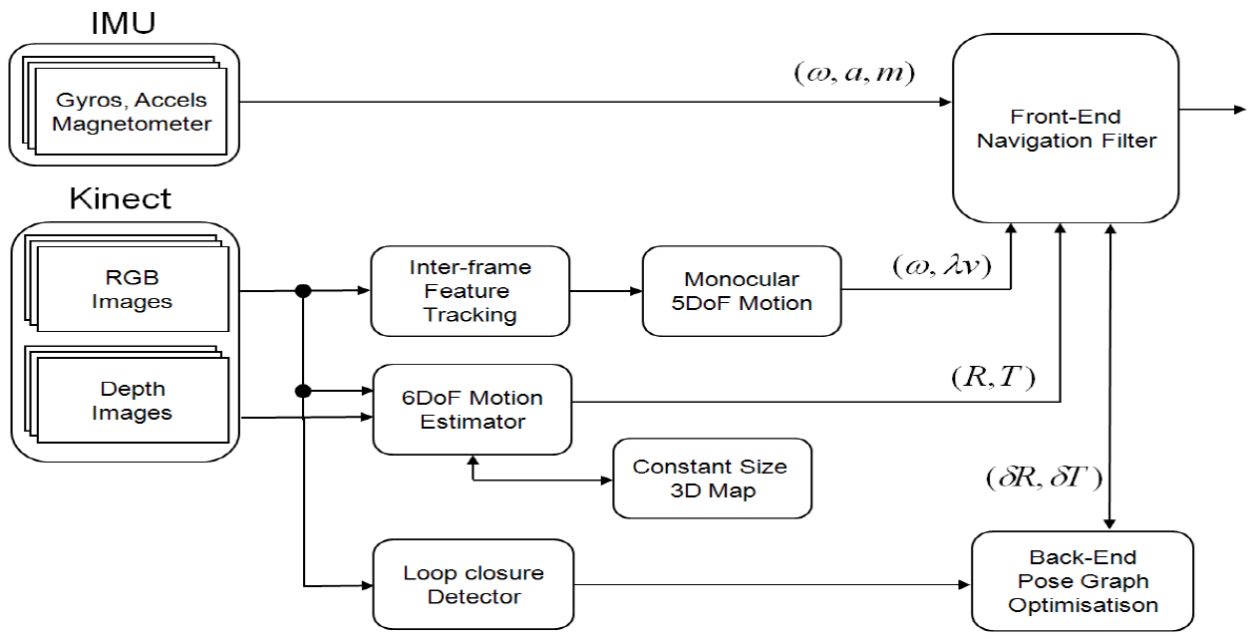
A loosely-coupled Inertial-Kinect odometry system architecture. RGB-D images are processed in a local Kinect odometry module that utilizes a window-based map for real-time processing. 2D RGB images are used for directional motion constraints and rotation rate and fused with inertial odometry within an extended Kalman filter. There is an off-board back-end SLAM that utilizes a keyframe-based graph SLAM to handle loop detection and update.

Figure 2 illustrates the architecture of the navigation system, which consists of a real-time, front-end odometry part and an off-board processed back-end SLAM part. An extended Kalman filter is designed using a loosely coupled integration. When 3D images are available from the Kinect sensor, a window-based, fixed-size map filter estimates the features’ positions to compute the full pose of the vehicle. The window-based map filters do not maintain the cross-correlations between the features and vehicle. Thus they are suboptimal but are computationally efficient and suitable for real-time estimation. When only 2D images are delivered, the translation information with scale ambiguity is converted as a directional motion constraint to aid the inertial outputs. The back-end SLAM is processed off-board and maintains keyframe images to detect loop closures and correction. The estimated pose of the vehicle is fed back to a flight controller, which subsequently generates control signals to the onboard microcontroller.

The paper is outlined as follows: Section 2 provides the literature review related to the RGB-D-based navigation and mapping. Section 3 provides the methods of inertial odometry, visual pose measurements with and without directional constraints, and the integration filter. Section 4 presents the experimental results and discussions from the indoor and outdoor environments, followed by conclusions.

## 2. Related Work

There exists a vast amount of literature on visual navigation and SLAM, and thus this review will focus on the RGB-D-related work and its integration with inertial sensors. The work by Huang et al. [10] uses full RGB-D information for 3D SLAM on aerial vehicles. It uses full color and depth information from a Kinect sensor to detect features from the gray-scale image and use their corresponding depths for the motion estimation. Keyframe-based feature matching is performed to estimate the final camera pose of the aerial vehicle in an indoor environment. The final smoothing is performed by graph-based optimization to build a globally consistent map. The use of depth-only information is proposed by Izadi et al. [11] for a hand-held scenario, utilizing the iterative closest point (ICP) method for structured indoor environments. Another work [12] focuses on the real-time performance in which an ICP and a constant size feature map are maintained for real-time implementation. Scherer et al. [13] also use depth information in the context of the mono-SLAM framework. Another work by [14] integrates the 3D visual odometry with the ICP-based SLAM approach.

The above mentioned RGB-D techniques heavily rely on the full depth information. The work of [15] addresses the depth dropout issue by solving the offline SLAM optimization problem for indoor conditions. Their work combines monocular and RGB-D measurements into a local map formation in an offline setting. The scale of the monocular camera is recovered in an offline scenario.

Considering the work in the visual-inertial domain, there exist two paradigms: tightly-coupled and loosely-coupled architecture. In the tightly-coupled paradigm, the work of [16,17,18,19,20] addresses the fusion of visual and inertial information using optimization or EKF-based SLAM. Ref. [16] applied the bundle adjustment technique for the visual-inertial odometry with an efficient loop-closure method. Ref. [17] applied a similar optimization method while eliminating any moving objects, such as pedestrians, improving the robustness of the visual odometry. Ref. [18] used the filtering approach exploiting the planar geometry of the ground plane. Although quite successful, these methods are computationally expensive as well as dependent on specific visual processing pipelines. Considering the rapid development of vision processing algorithms, the integration algorithms need to be revised accordingly. Any bundle adjustment (e.g., VINS mono, DUI-VIO) or depth estimation methods (inverse depth parameterization) can cause drift in the IMU solution during the process. Other papers mentioned exploit certain geometry such as planar ground or moving object elimination, which are different to our focus.

An alternative architecture is a loosely coupled method in which the visual and inertial information is treated as a separate entity, and visual constraints are used to update and aid the inertial sensor [21]. The gyro information is also used to help the RGB-D pose estimator as in [22,23], in which gyroscopes are used to estimate the rotation of the cameras, or as a prior to the ICP algorithm. Ref. [4] addresses the degeneracy problem of the IMU-Kinect sensor utilizing the indoor plane features from the camera. These Kinect-based approaches either work indoors or require a structured environment. Refs. [24,25] uses an indirect Kalman filter that is based on the errors in the estimated measurement instead of the direct measurements from the camera and IMU systems. The work estimates the scale of the monocular camera motion estimate in the filter with an assumption of a smoothly changing scale of the scene. Learning techniques can provide a good alternative to fill the gaps in the depth image. There have been several supervised/semi-supervised depth mappings mostly in road environments, and it would be interesting to see their performance in outdoor/forest environments, which are unstructured and irregular.

Our work follows the loosely-coupled approach with direct-filter implementation (the advantage of the loosely-coupled system is constant-time processing and a modular implementation). Using the concept of visual directional constraints, we avoid the explicit estimation of the scale in integrating the monocular and IMU. The proposed framework consists of two modules, a front-end EKF-based odometry system, and a back-end module based on pose-graph optimization for global consistency. The map is not maintained in the EKF, hence resulting in the loosely coupled architecture. The benefit is the system becomes more modular, and other vision algorithms can be effectively incorporated.

## 3. Methods

### 3.1. Inertial Odometry

The inertial odometry model consists of the kinematic equations of an inertial navigation system driven by the IMU measurements, which are the specific force (or the sum of the dynamic acceleration and gravity) and angular rate. The position ($P^{n}$), velocity ($V^{n}$), and Euler angles ($\Psi^{n}$) of the vehicle are defined with respect to a local tangent, local-fixed navigation frame, and evolve as

(1) $$\begin{array}{cc} {\dot{P}}^{n} & =V^{n}\\ {\dot{V}}^{n} & =R_{b}^{n}\left(f^{b}-b_{a}^{b}\right)-2\omega_{ie}^{n}\times V^{n}+g^{n}\left(P^{n}\right)\\ {\dot{\Psi }}^{n} & =E_{b}^{n}\left(\omega^{b}-b_{g}^{b}\right), \end{array}$$

where

- $\omega_{ie}^{n}$ is the Earth rotation rate in the navigation frame;
- $g^{n}\left(P^{n}\right)$ is the acceleration due to gravity;
- $f^{b}$ is the accelerometer measurement in the body frame;
- $\omega^{b}$ is the gyroscope measurement in the body frame;
- $b_{a}^{b}$ is the accelerometer bias in the body frame;
- $b_{g}^{b}$ is the gyroscope bias in the body frame;
- $R_{b}^{n}$ is a direction cosine matrix transforming a vector from body to navigation frame
  $$\begin{array}{c} R_{b}^{n}=\left[\begin{array}{ccc} c_{\theta }c_{\psi } & -c_{\phi }s_{\psi }+s_{\phi }s_{\theta }c_{\psi } & s_{\phi }s_{\psi }+c_{\phi }s_{\theta }c_{\psi }\\ c_{\theta }s_{\psi } & c_{\phi }c_{\psi }+s_{\phi }s_{\theta }s_{\psi } & -s_{\phi }c_{\psi }+c_{\phi }s_{\theta }s_{\psi }\\ -s_{\theta } & s_{\phi }c_{\theta } & c_{\phi }c_{\theta } \end{array}\right] \end{array}$$
- $E_{b}^{n}$ is a matrix transforming a body rate to an Euler angle rate.
  $$\begin{array}{c} E_{b}^{n}=\left[\begin{array}{ccc} 1 & s_{\phi }t_{\theta } & c_{\phi }t_{\theta }\\ 0 & c_{\phi } & -s_{\phi }\\ 0 & s_{\phi }/c_{\theta } & c_{\phi }/c_{\theta } \end{array}\right], \end{array}$$
  where $s_{\left(\cdot \right)}$, $c_{\left(\cdot \right)}$, and $t_{\left(\cdot \right)}$ are shorthand notations for sin(·), cos(·), and tan(·), respectively.

Although the Euler angles have a singularity problem when the pitch angle approaches $90^{\circ }$, it rarely happens in most drone operational scenarios. Thus, due to the simplicity compared to other representations such as the quaternion, the Euler angles are adopted in this work.

### 3.2. Visual Pose Measurement

**Figure 3:**
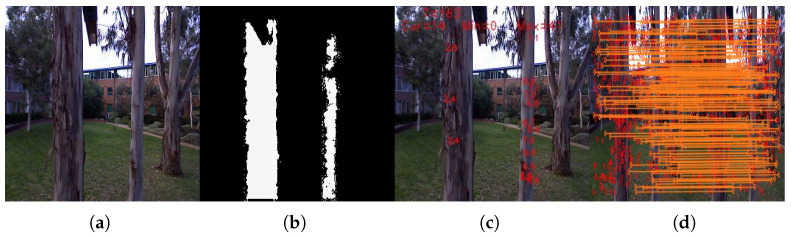
Feature detection and matching process. (**a**) Input image. (**b**) Corresponding depth image. (**c**) Selected image features that have corresponding depths. (**d**) Feature matching between two consecutive images using SURF descriptors followed by RANSAC.

Figure 3 shows that a pipeline of Kinect image processing is performed to extract the visual features and match them across frames. In this work, Harris corners are used on the gray-scale image. If the corner features do not have the corresponding depth information, they are discarded from the feature list. Speed-up robust features (SURF) descriptors are used for the feature matching purpose. The Kinect odometry module consists of two parts: 6DOF and 5DOF pose processing modules. The full 6DOF poses are computed when the depth information is available from the Kinect sensor. When the depth dropouts occur, then the 5DOF poses are computed.

#### 6DOF Pose Measurement

The 6DOF pose measurement is the rigid-body transformation (R,P) of the camera from its original pose and is obtained in two steps. First, an initial pose is computed using the closed-form solution from the point clouds as in [26]. It is then used to run a weighted-ICP (iterative closest point) for fine refinement.

The spatial location of the feature in the pixel coordinates with raw depth gives $\left(u,v,d\right)\in R^{3}$, which can be converted into a 3D Euclidian feature position, $\left(x,y,z\right)\in R^{3}$, relative to the camera. The mapping function g:(u,v,d)→(x,y,z) becomes:

(2) $$\begin{array}{c} \begin{array}{c} x=\frac{z}{f}\left(u-u_{0}\right),\;y=\frac{z}{f}\left(v-v_{0}\right),\;z=\frac{f}{d}L, \end{array} \end{array}$$

where *f* is the camera focal length, $\left(u_{0},v_{0}\right)$ is the center of the image, and *L* is the baseline length between the infrared emitter and the receiver in the Kinect sensor. The related covariance matrix W of the transformed Euclidian 3D position can be computed using a Jacobian of the mapping function, assuming independent noise in pixel and depth measurements:

(3) $$\begin{array}{c} W=J\left(\begin{array}{ccc} \sigma_{u}^{2} & 0 & 0\\ 0 & \sigma_{v}^{2} & 0\\ 0 & 0 & \sigma_{d}^{2} \end{array}\right)J^{T},\;\mathrm{with}\;J=\frac{\partial g(x,y,z)}{\partial (u,v,d)}. \end{array}$$

The 3D features are declared as a map (M) defined in the local navigational frame. All the subsequent feature measurement data (D) are matched with the existing map features using the SURF descriptors. The comparing score is based on the sum-of-absolute-difference and if it is within a specified threshold then it is declared a matched-pair. As this matching can still lead to wrong matches, RANSAC is used to remove the outliers during the optimization:

(4) $$\begin{array}{c} \underset{R,P}{\arg\min}\left(\frac{1}{N}\sum_{i\in A}c_{i}{∥M_{i}-\left(R\;D_{i}+P\right)∥}_{W_{i}}^{2}\right), \end{array}$$

where *i* stands for the index of inlier feature-set *A*, and $c_{i}$ is the correspondence with W being the weighting matrix from (3).

A ring buffer of features is maintained to track the locally tracked features, while the global keyframe map is retained in the pose-graph module as discussed in Section 3.5. The features within a predefined Euclidean vicinity are declared as update points, whereas others are declared as new points. The existing points are updated using a weighted averaging method. If the limit of the ring buffer is reached, then the old features are deleted.

### 3.3. 5DOF Measurement Using Directional Constraints

The 2D image processing pipeline is similar to the 3D case except the local feature map is not utilized. The rotation (R) and translation (λP) are estimated using the standard 5-point visual odometry algorithm together with RANSAC. Using the sampling time, the motion between two consecutive images is converted to the rotational rate and translational velocity (up to scale) (ω,λV). In order to integrate these motion estimates with the inertial sensor (which operates in metric space), the translational velocity is further converted into a *unit directional constraint* in the body frame $U^{b}$. This constraint can also be related to the inertial odometry. That is, the unit velocity in the body frame can be obtained from the unit velocity in navigation frame $U^{b}={\left[R_{b}^{n}\right]}^{T}V^{n}/\left|\right|V^{n}\left|\right|$, yielding, -4.6cm0cm

(5) $$\begin{array}{c} \left[\begin{array}{c} U_{x}^{b}\\ U_{y}^{b}\\ U_{z}^{b} \end{array}\right]=\frac{1}{\sqrt{V_{N}^{2}+V_{E}^{2}+V_{D}^{2}}}\left[\begin{array}{c} c_{\theta }c_{\psi }V_{N}+c_{\theta }s_{\psi }V_{E}-s_{\theta }V_{D}\\ \left(-c_{\phi }s_{\psi }+s_{\phi }s_{\theta }c_{\psi }\right)V_{N}+\left(c_{\phi }c_{\psi }+s_{\phi }s_{\theta }s_{\psi }\right)V_{E}+s_{\phi }c_{\theta }V_{D}\\ \left(s_{\phi }s_{\psi }+c_{\phi }s_{\theta }c_{\psi }\right)V_{N}+\left(-s_{\theta }c_{\psi }+c_{\phi }s_{\theta }s_{\psi }\right)V_{E}+c_{\phi }c_{\theta }V_{D} \end{array}\right] \end{array}$$

If the vehicle motion is constrained to the ground, this is similar to the non-holonomic motion constraint. For example, the tangential components of the velocity $\left(V_{y}^{b}=0,V_{z}^{b}=0\right)$ become zero in the body frame, assuming no side skidding. In a general 3D case, such as for a flying vehicle, this constraint does not hold.

The concept of the *directional constraints* naturally extends this non-holonomic motion constraint to the visual velocity in which the lateral image velocities of the visual motion are treated as zero. The key benefit of this concept is the undelayed aiding of IMU outputs without requiring 3D information of the features or map. However, the longitudinal image velocity is unobservable and thus requires additional depth information, which is delivered from a pose-graph SLAM module.

### 3.4. Integration Filter with Directional Constraints

An extended Kalman filter is designed to integrate the inertial and Kinect measurements in a loosely-coupled integration architecture. After discretization, the state Equation (1) and the observation equation with directional constraints (5) become:

(6) $$\begin{array}{cc} x(k) & =f(x(k-1),u(k-1),w(k-1)) \end{array}$$

(7) $$\begin{array}{cc} z(k) & =h(x(k),v(k)), \end{array}$$

where x(k),u(k),and z(k) are the state vector, control input, and measurement vector at time step *k*, respectively. w(k) and v(k) are the process and observation noise, which have zero means and strength matrices Q and R.

Given the models, the estimate of the state $\hat{x}\left(k|k\right)$ and covariance P(k|k) can be recursively computed within the filter. First, the predicted state and covariance become:

(8) $$\begin{array}{cc} \hat{x}\left(k|k-1\right) & =f(\hat{x}\left(k-1|k-1\right),u\left(k-1\right),0) \end{array}$$

(9) $$\begin{array}{cc} P(k|k-1) & =\nabla f_{x}P\left(k-1|k-1\right)\nabla f_{x}^{T}+\nabla f_{u}Q\nabla f_{u}^{T}, \end{array}$$

where ∇ represents the gradient operator.

The switching criteria between RGB-D and RGB measurements are based upon the availability of depth features and their spatial distribution. If the number of features is uniformly distributed over the image and depth features are available, then the RGB-D measurements are used to update the EKF filter. Otherwise, the monocular directional constraints are used for the filter update. The uncertainty of the measurements is scaled directly with the number of inliers in order to gauge the quality of motion estimates. In order to cater to the measurement delay in the vision processing pipeline, we maintain a timestamp of each predicted state (from EKF) in the ring buffer. Whenever the Kinect measurements (RGB-D or visual constraints) are available, the past EKF state is retrieved/updated accordingly, and the corrected state is then propagated to the current state. When a measurement is available, innovation and its covariance are calculated as follows:

(10) $$\begin{array}{cc} \nu (k) & =z\left(k\right)-h(\hat{x}\left(k|k-1\right),0) \end{array}$$

(11) $$\begin{array}{cc} S(k) & =\nabla h_{x}P\left(k|k-1\right)\nabla h_{x}^{T}+\nabla h_{v}R\nabla h_{v}^{T}. \end{array}$$

Then the state estimate and its covariance are updated:

(12) $$\begin{array}{cc} \hat{x}\left(k|k\right) & =\hat{x}\left(k|k-1\right)+K\left(k\right)\nu \left(k\right) \end{array}$$

(13) $$\begin{array}{cc} P(k|k) & =P\left(k|k-1\right)-K\left(k\right)S\left(k\right)K{\left(k\right)}^{T}, \end{array}$$

with a Kalman gain matrix:

(14) $$\begin{array}{c} K\left(k\right)=P\left(k|k-1\right)\nabla h_{x}^{T}S^{-1}\left(k\right). \end{array}$$

### 3.5. Pose-Graph Optimization

As a back-end module, a keyframe-based pose-graph SLAM is applied to constrain the inertial-Kinect odometry further. Keyframes are selected from the Kinect measurements using the threshold on the accumulated motion estimates. Their corresponding pose/state from the EKF filter is passed to the pose-graph optimizer (only for selected keyframes). A new edge constraint is added to the pose-graph when a loop is detected using the SURF descriptor matching between the keyframes and the current image frame. Subsequently, the graph is optimized, and on convergence, the filter state (for the respective timestamp of the keyframe) is updated in the ring buffer. The corrected state is then propagated to the current EKF state to minimize the effect of drift.

### 3.6. Observability of the System

The extended Kalman filter designed in the previous section integrates the 3D or 2D visual measurements depending on the availability of the depth information. If the directional constraints are incorporated as in Equation (5), it is clear that the velocity vector becomes partially observable due to the unknown velocity scale λ. In addition, the velocity estimated from the IMU requires integration of the acceleration and thus does not increase the observability of the velocity state. If we use an instantaneous coordinate system of the motion (*m*) and express the velocity along the axial (‖) and normal (⊥) directions, the velocity vector $V^{m}=V_{\|}^{m}+V_{⊥}^{m}=V_{\|}^{m}$, as the tangential velocity components are zero. The axial velocity component can be made observable from the 3D measurements with depth information, which effectively computes the scale of the translation and thus the velocity. Please note that the unknown velocity scale can be estimated within the EKF, as in the popular inverse-depth parametrization approaches. However, the predicted velocity from the IMU is also unobservable due to the integration process, and thus the estimated scale suffers from drifting, causing the so-called scale drift problem. It can only be properly estimated from the 3D measurements as in our work or the loop-closures in SLAM.

## 4. Results and Discussion

### 4.1. Depth Calibration

The Kinect sensor used is reasonably well-calibrated from the factory settings. However, the raw range output is expressed as inverse disparity, not actual depth, thus requiring further calibration. We adopted the methods from [27], in which a checker-board is used for intrinsic/extrinsic parameter estimation using bundle adjustment-based refinement. We estimate the depth provided from the Kinect sensor for a region of interest (where the object is present) and average it. After the calibration, the depth with respect to the ground truth shows less than 1% error for up to a 3-m range, showing consistent depth results. After the depth calibration, the RGB camera is calibrated using a standard camera method. Finally, the calibration between the vision and inertial sensor is performed using the method proposed by [28], where the rotational misalignment is estimated by using the direction of gravity (from the accelerometers) and the camera’s vertical orientation.

### 4.2. Indoor Experiment

**Figure 4:**
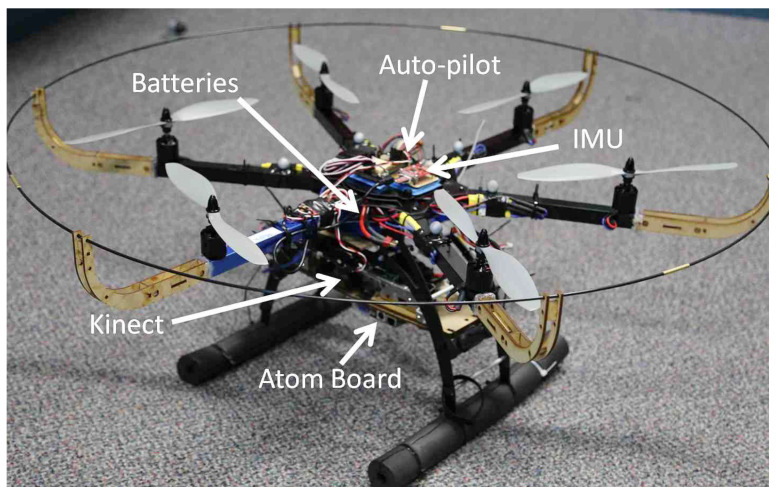
A hexacopter platform equipped with Kinect/IMU sensors and an Atom processor.

A hexacopter platform is developed, which is equipped with a low-cost IMU with a 38 Hz data rate and a Kinect RGB-D sensor at 22 Hz, as shown in Figure 4. To evaluate the performance in an indoor environment, a Vicon motion capture system is utilized. A dual-core Atom embedded computer mounted on the platform collects and processes the data, running under the Robot Operating System (ROS). All data are timestamped for the synchronization, and ring-buffers are also used to handle the time difference between the acquisition and processing time. The hexacopter autopilot system is modified to accommodate the position control commands from the Atom processor. A cascaded PID position controller running at 50 Hz generates the waypoints and hovering commands using the Inertial-Kinect odometry outputs.

**Figure 5:**
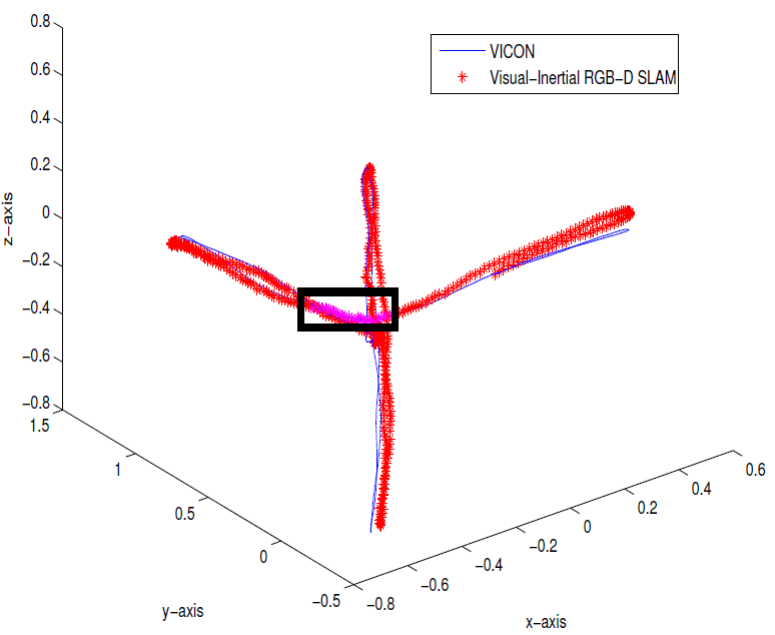
Indoor results of real-time Inertial-Kinect estimated trajectory (in red) compared with the Vicon ground truth (in blue). Directional constraint updates are simulated and shown in the rectangular box.

**Figure 6:**
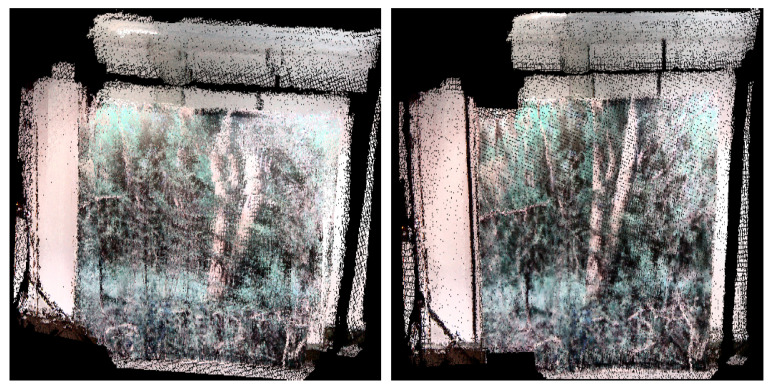
Indoor results: before (**left**) and after (**right**) pose-graph optimization where the room wall was textured with forest-like images.

To verify the method, 900 Kinect frames and 1501 IMU data packets were collected from an indoor environment. To simulate the depth dropouts, some of the depth data were discarded to verify the proposed approach. The estimated pose from the proposed method was compared against the ground truth data from Vicon, as shown in Figure 5. The trajectory shows the take-off and lateral movements of the hexacopter platform, and the dropouts are shown in a rectangular box. The errors were computed using the ground truth in terms of root-mean-square error (RMSE). Table 1 summarizes the performance showing that the RMSE is less than 0.2 m and $0.5^{\circ }$, and there is an improved performance closely resembling the ground truth. Figure 6 also confirms the consistency of the system, showing a visibly consistent 3D map after the pose-graph SLAM optimization.

### 4.3. Public Indoor Dataset

We also tested the proposed method for the publicly available dataset (fr1/desk, fr2/desk and fr1/room) from the University of Freiburg [30] to compare the performance of the Inertial-Kinect solutions. Each dataset comes with an accurate ground truth captured by external motion capture systems (Vicon). Table 2 summarizes the results on the relative pose error (RPE) for more datasets (fr1/xyz and fr2/xyz), confirming accurate estimates compared to the ground-truth data. Table 3 compares the proposed method with the state-of-the-art SLAM methods in terms of the absolute trajectory error (ATE): robust edge-based VO (REVO) key frame (KF) [31], REVO frame-to-frame (FF) [31], FOVIS (an ROS module for visual odometry) [10], and dense visual odometry [14]. The comparison confirms that our proposed method performs better or with competitive accuracy compared to those methods.

### 4.4. Outdoor Experiment

**Figure 7:**
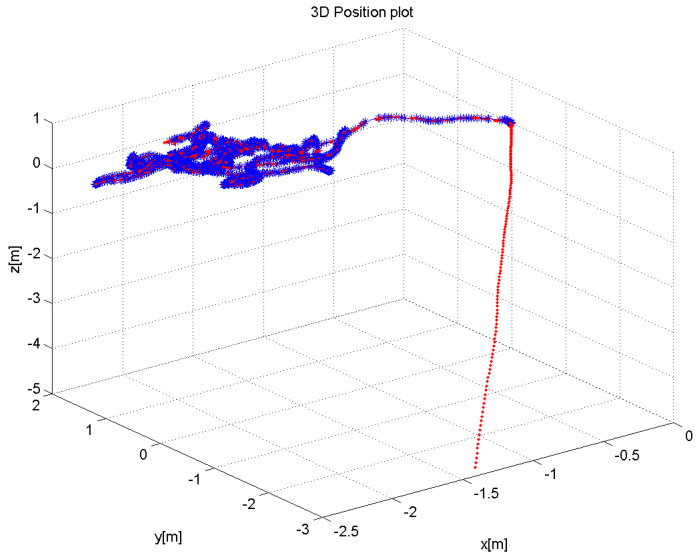
Estimated 3D flight trajectory of the hexacopter for outdoor sequence (in blue) and inertial output (in red). The Kinect sensor was activated after the take-off, showing some drift in the inertial output.

**Figure 8:**
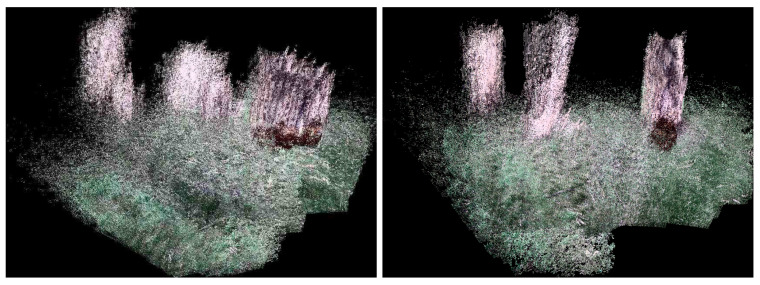
Outdoor mapping results before (**left**) and after (**right**) the pose-graph optimization showing more consistent 3D map after the optimization.

Currently, to our knowledge, there is no public 3D dataset from a forest-like environment. Therefore outdoor flight tests were performed in a cluttered tree environment. The average flight height was 10 m above the ground, and the maximum ground speed was approximately 5–7 m/s. The environment was challenging due to the absence of GPS position sensing due to the tree canopy. An area of 10m×12m was explored by a manual pilot mode collecting 1701 RGB-D Kinect frames in which 240 data frames lacked depth information due to the depth dropout. Figure 7 shows the 3D trajectory of the aerial vehicle, which was estimated in real-time from the onboard computer. Figure 8 also shows the pose-graph optimization results processed on an off-board laptop, which also shows an input image, a 3D depth map used in the Kinect odometry. It can be seen that the pose graph optimization makes the global keyframe maps visibly more consistent. As there is no absolute GPS information available for the comparison, the normalized innovation sequences were used to check the filter consistency, showing that most of the sequence falls within the 95.5% confidence interval. The results confirm that the proposed Inertial-Kinect algorithm is capable of estimating the vehicle states in a challenging outdoor environment. The Kinect processing time is also summarized in Table 4, having less than 100ms processing time and thus the real-time capability of the method.

However a high-speed camera with fast optical-flow algorithms can also be utilized to improve the navigational accuracy, thanks to the loosely coupled integration of the vision processing module. The real-time management of the estimator is also crucial for the control and guidance of the vehicle. Currently, the 10 Hz pose output rate is adequate for the high-level control of the vehicle, thanks to the fast internal angular stabilization within the drone.

## 5. Conclusions

An Inertial-Kinect integration framework was presented, which fuses an IMU odometry and Kinect odometry in a loosely coupled EKF integration architecture. The Kinect odometry system computes the full 6DOF, or partial 5DOF poses depending on the depth availability. An efficient and fixed-size local feature map is maintained to calculate the full Kinect odometry. When depth dropouts occur, the visual translation is used as a directional motion constraint. The lateral image velocity components become zero, which enables a seamless aiding of IMU errors without delay. The back-end SLAM module performs the pose-graph optimization detecting the loop closures and further correcting the IMU errors. Indoor and outdoor flight results demonstrate the robustness of the proposed approach in challenging outdoor environments. Future work will involve combining the Inertial-Kinect odometry outputs and path-planning algorithms with exploring the outdoor settings.

## Figures and Tables

**Table 1:** Evaluation of proposed approach against Vicon outputs (RMSE error).

	$P_{x}(m)$	$P_{y}(m)$	$P_{z}(m)$	ϕ (${}^{\circ }$)	θ (${}^{\circ }$)	ψ (${}^{\circ }$)
Monocular Visual-Inertial [29]	2.61	3.13	1.79	$1.07^{\circ }$	$1.01^{\circ }$	$0.81^{\circ }$
Inertial-Kinect w/o directional constraints	2.43	1.75	2.66	$3.84^{\circ }$	$2.70^{\circ }$	$2.14^{\circ }$
Inertial-Kinect with directional constraints	0.05	0.04	0.19	$0.40^{\circ }$	$0.04^{\circ }$	$0.48^{\circ }$

**Table 2:** Relative pose error (RPE) evaluation of proposed approach against Vicon outputs.

Dataset	Trans (m/s)	Max Trans (m/s)	Rotation (${}^{\circ }$/s)	Max Rotation (${}^{\circ }$/s)
fr1/xyz	0.120	0.053	1.342	5.415
fr1/desk	0.011	0.372	4.219	9.173
fr2/xyz	0.301	0.017	0.321	4.310
fr2/desk	0.147	0.172	3.102	3.999

**Table 3:** Comparison of median/maximum values of ATE with state-of-the-art algorithms.

Algorithm	fr1 Desk		fr2 Desk		fr1 Room	
	Median	Max	Median	Max	Median	Max
RGB-D SLAM [14]	0.068	0.231	0.118	0.346	0.152	0.419
Monocular SLAM [29]	0.931	1.763	0.982	1.621	2.531	0.792
REVO-KF [31]	-	0.547	-	0.095	-	0.288
REVO-FF [31]	-	0.186	-	0.329	-	0.305
FOVIS [10]	0.221	0.799	0.112	0.217	−0.238	0.508
Proposed Approach	0.024	0.214	0.012	0.092	0.133	0.317

**Table 4:** Kinect processing time on an embedded computer (Kinect update rate is 10 Hz).

Module	Processing Time per Frame (ms)
Data acquisition	08.60
Feature detection and matching	43.17
Motion estimation	28.92
Pose update	19.05

## References

[1] Zhang H., Ye C. DUI-VIO: Depth Uncertainty Incorporated Visual Inertial Odometry based on an RGB-D Camera. Proceedings of the IEEE/RSJ International Conference on Intelligent Robots and Systems.

[2] Li H., Wen I.D.X., Guo H., Yu M. (2018). Research into Kinect/Inertial Measurement Units Based on Indoor Robots. Sensors.

[3] Chai W., Chen C. Enhanced Indoor Navigation Using Fusion of IMU and RGB-D Camera. Proceedings of the International Conference on Computer Information Systems and Industrial Applications (CISIA).

[4] Cho H., Yeon S., Choi H., Doh N. (2018). Detection and Compensation of Degeneracy Cases for IMU-Kinect Integrated Continuous SLAM with Plane Features. Sensors.

[5] Qayyum U., Kim J. Inertial-Kinect Fusion for Outdoor 3D Navigation. Proceedings of the Australasian Conference on Robotics and Automation.

[6] Dai X., Mao Y., Huang T., Li B., Huang D. Navigation of Simultaneous Localization and Mapping by Fusing RGB-D Camera and IMU on UAV. Proceedings of the CAA Symposium on Fault Detection, Supervision and Safety for Technical Processes.

[7] Diel D.D., DeBitetto P., Teller S. Epipolar Constraints for Vision-Aided Inertial Navigation. Proceedings of the Seventh IEEE Workshops on Applications of Computer Vision.

[8] Fang W., Zheng L. (2018). Rapid and robust initialization for monocular visual inertial navigation within multi-state Kalman filter. Chin. J. Aeronaut..

[9] Pire T., Fischer T., Castro G., Cristóforis P.D., Civera J., Berlles J.J. (2017). S-PTAM: Stereo Parallel Tracking and Mapping. Robot. Auton. Syst..

[10] Huang S., Bachrach A., Henry P., Krainin M., Maturana D., Fox D., Roy N. (2011). Visual Odometry and Mapping for Autonomous Flight Using an RGB-D Camera. Robotics Research, Proceedings of the 15th International Symposium on Robotics Research (ISRR), Flagstaff, AZ, USA, 28 August–1 September 2011.

[11] Izadi S., Kim D., Hilliges O., Molyneaux D., Newcombe R., Kohli P., Shotton J., Hodges S., Freeman D., Davison A. KinectFusion: Real-Time 3D reconstruction and interaction using a moving depth camera. Proceedings of the 24th Annual ACM Symposium on User Interface Software and Technology.

[12] Dryanovski I., Valenti R., Xiao J. Fast Visual Odometry and Mapping from RGB-D Data. Proceedings of the IEEE International Conference on Robotics and Automation (ICRA).

[13] Scherer S.A., Dube D., Zell A. Using depth in visual simultaneous localization and mapping. Proceedings of the Robotics and Automation (ICRA).

[14] Whelan T., McDonald J., Johannsson H., Kaess M., Leonard J. Robust Real-Time Visual Odometry for Dense RGB-D Mapping. Proceedings of the IEEE International Conference on Robotics and Automation (ICRA).

[15] Hu G., Huang S., Zhao L., Alempijevic A., Dissanayake G. A robust RGB-D SLAM algorithm. Proceedings of the IEEE/RSJ International Conference on Intelligent Robots and Systems (IROS).

[16] Qin T., Li P., Shen S. (2018). VINS-Mono: A Robust and Versatile Monocular Visual-Inertial State Estimator. IEEE Trans. Robot..

[17] Fu D., Xia H., Qiao Y. (2021). Monocular Visual-Inertial Navigation for Dynamic Environment. Remote Sens..

[18] Yang Y., Geneva P., Zuo X., Eckenhoff K., Liu Y., Huang G. Tightly-Coupled Aided Inertial Navigation with Point and Plane Features. Proceedings of the International Conference on Robotics and Automation.

[19] Jones E., Soatto S. (2011). Visual-inertial navigation, mapping and localization: A scalable real-time causal approach. Int. J. Robot. Res..

[20] Mourikis I., Roumeliotis S. A multistate constraint Kalman filter for vision-aided inertial navigation. Proceedings of the IEEE International Conference on Robotics and Automation (ICRA).

[21] Konolige K., Agrawal M., Sola J. Large scale visual odometry for rough terrain. Proceedings of the International Symposium on Research in Robotics (ISRR).

[22] Bouvrie B. (2011). Improving RGBD Indoor Mapping with IMU Data. Master’s Thesis.

[23] Ovren H., Forssen P., Tornqvist D. Why Would I Want a Gyroscope on my RGB-D Sensor?. Proceedings of the IEEE Winter Vision Meetings, Workshop on Robot Vision (WoRV13).

[24] Weiss S., Siegwart R. Real-Time Metric State Estimation for Modular Vision-Inertial Systems. Proceedings of the IEEE International Conference on Robotics and Automation (ICRA).

[25] Nuetzi G., Weiss S., Scaramuzza D., Siegwart R. (2011). Fusion of IMU and Vision for Absolute Scale Estimation in Monocular SLAM. J. Intell. Robot. Syst..

[26] Horn B. (1987). Closed-form solution of absolute orientation using unit quaternions. J. Opt. Soc. Am. A.

[27] Herrera C., Kannala D., Heikkila J. (2012). Joint depth and color camera calibration with distortion correction. IEEE Trans. Pattern Anal. Mach. Intell..

[28] Kelly J., Sukhatme S. (2011). Visual-inertial sensor fusion:localization mapping and sensor-to-sensor self-calibration. Int. J. Robot. Res..

[29] Qayyum U., Kim J. Seamless aiding of inertial-slam using Visual Directional Constraints from a monocular vision. Proceedings of the Intelligent Robot Systems (IROS).

[30] Sturm J., Engelhard N., Endres F., Burgard W., Cremers D. A benchmark for the evaluation of RGB-D SLAM systems. Proceedings of the IEEE/RSJ International Conference on Intelligent Robots and Systems (IROS).

[31] Khoshelham K., Elberink S.O. (2012). Accuracy and Resolution of Kinect Depth Data for Indoor Mapping Applications. Sensors.

